# Importance of intellectual property generated by biomedical research at universities and academic hospitals

**DOI:** 10.18053/jctres.03.201702.005

**Published:** 2017-05-24

**Authors:** Joris J. Heus, Elmar S. de Pauw, Leloux Mirjam, Morpurgo Margherita, Hamblin Michael R, Heger Michal

**Affiliations:** 1 Innovation Exchange Amsterdam (IXA) Office AMC, Academic Medical Center, University of Amsterdam, Meibergdreef 9, 1105 AZ Amsterdam, the Netherlands; 2 Innovation Exchange Amsterdam (IXA) Office UvA-HvA, University of Amsterdam, Science Park 904, Amsterdam, the Netherlands; 3 Department of Pharmaceutical and Pharmacological Sciences, University of Padova, Padova, Italy; 4 Technology Transfer Office, University of Padova, Padova, Italy; 5 Wellman Center for Photomedicine, Massachusetts General Hospital, Boston, Massachusetts, United States; 6 Department of Dermatology, Harvard Medical School, Boston, Massachusetts, United States; 7 Harvard-MIT Division of Health Sciences and Technology, Cambridge, Massachusetts, United States; 8 Department of Experimental Surgery, Academic Medical Center, University of Amsterdam, Amsterdam, the Netherlands

**Keywords:** invention, technological, innovation, medical, pharmaceutical, research, patents, protection, commercialization

## Abstract

Biomedical research has many different facets. Researchers and clinicians study disease biology and biochemistry to discover novel therapeutic targets, unravel biochemical pathways and identify biomarkers to improve diagnosis, or devise new approaches to clinically manage diseases more effectively. In all instances, the overall goal of biomedical research is to ensure that results thereof (such as a therapy, a device, or a method which may be broadly referred to as “inventions”) are clinically implemented. Most of the researchers’ efforts are centered on the advance of technical and scientific aspects of an invention. The development and implementation of an invention can be arduous and very costly. Historically, it has proven to be crucial to protect intellectual property rights (IPR) to an invention (i.e., a patent) to ensure that companies can obtain a fair return on their investment that is needed to develop an academic invention into a product for the benefit of patients. However, the importance of IPR is not generally acknowledged among researchers at academic institutions active in biomedical research. Therefore this paper aims to (1) raise IP awareness amongst clinical and translational researchers; (2) provide a concise overview of what the patenting trajectory entails; and (3) highlight the importance of patenting for research and the researcher.

**Importance for patients**: Adequate patent protection of inventions generated through biomedical research at academic institutions increases the probability that patients will benefit from these inventions, and indirectly enables the financing of clinical studies, mainly by opening up funding opportunities (e.g. specific grants aimed at start-ups, pre-seed and seed capital) that otherwise would not be accessible. As a consequence, patented inventions are more likely to become clinically tested and reach the market, providing patients with more treatment options.

## 1 Background

The majority of biomedical researchers at universities and academic medical centers work on projects ultimately intended to benefit patients. Whether the project entails the elucidation of mechanisms in different diseases or the development of novel medical devices or therapeutics, it is the eventual clinical problem that initially motivates the researcher. An easy yet illustrative example is the problem of cancer. Due to its status as a “dread disease,” cancer is a top-priority medical problem that is well-funded and extensively researched at multiple levels. Some investigators study cancer biology and biochemistry in the hope to discover novel therapeutic targets, others investigate how to improve cancer diagnosis and study epidemiology, while another group of researchers focuses on devising new clinical approaches to pinpoint and eradicate tumors. In the grand scheme of research, the overall goal of any of these projects is to ensure that the information, drug, or device is eventually commercialized in order for it to achieve clinical implementation.

An important fact in this grand scheme is that the costs associated with clinical application and testing of a drug or device typically amount up to many millions (if not billions) of dollars. Pharmaceutical and medical device companies operate on business models that account for these costs, whereas universities and affiliated hospitals have a different business model. Universities and academic hospitals mainly use their primary source of income that is received from tuition costs and patient care revenue to cover general business operations and employee costs, leaving few remaining funds to invest in product development and clinical trials.

Instead, universities and academic hospitals commonly make use of grants acquired from secondary (industry) or tertiary sources (government) to fund research projects. Such funding however, is generally insufficient to develop a clinical product.

**Figure 1:**
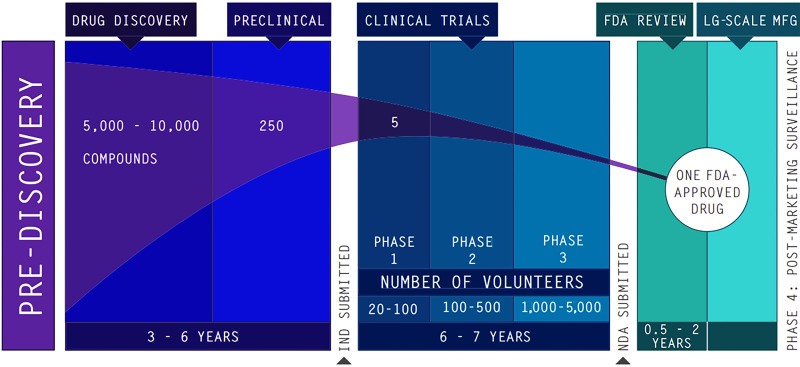
Typical pharmaceutical product development timeline and num of compounds needed in the different phases to obtain one FDA-approved drug. Adapted from [1].

A solution to this problem is to combine academic research with elements of the corporate business model, illustrated in Figure 1 for pharmaceutical product development. This means that intellectual property (IP) rights should be established on inventions that have been conceptualized during the course of the research project. These patented inventions may be further exploited in R&D trajectories by existing (pharma or biotech) companies or within a university spin-off or start-up company, separate from the university, but often still connected with the inventors. Such an R&D trajectory becomes attractive for funding from pharma companies or for infusions of pre-seed capital (up to ~250K Euro, often provided by a government-related fund or university holding company) and seed capital (~500K-1M Euro, provided from angel investors or early stage venture capital firms). Often, such external funding is sufficient to cover the large expenses of pre-clinical development and clinical trials.

To fully take advantage of this stream of research funding, most academic institutions and affiliated hospitals have established a knowledge transfer office (KTO), in the US also known as Innovations Departments. In addition, some national governments have set up monetary support programs to enable researchers to obtain proof-of-concept data e.g., to facilitate patented inventions and to stimulate the discoverers to investigate the possibility of starting a spin-off company. These programs, which have gained momentum in the last decade, have matured and as a result many academic institutions now boast a portfolio of spin-off companies.

A significant bottleneck that still remains is that academic researchers often do not have the awareness, business mindset, or in-depth knowledge of IP-related issues to efficiently proceed with patenting their invention in addition to publishing their data. The problem here is that a public disclosure of research findings may destroy the patentability of any invention arising from data contained in the publication (i.e. the invention is not considered to be novel anymore), and therefore reduces the possibility that such invention will ultimately benefit the end-users of their research (i.e., the patients). Especially in the pharmaceutical business, decisions on whether or not to develop a certain product heavily depend on the existence of a strong IP position, as the chances of generating a good return on investment is low when competitors cannot be blocked. No investor will finance a development project if the underlying IP has been lost by a too early disclosure. As a result, potentially good inventions are lost for patients, because of the lack of patent protection leading to weak commercial prospects.

The above situation can be avoided. If scientists would be (made) more aware of the basic rules dictating the process of IP protection, it would be easier for them to adopt simple practices that allow free scientific exchange, while providing proper background for potential business exploitation (see also section 3.2.1).

The goal of this paper is to: (1) increase IP awareness amongst clinical and translational researchers; (2) provide a concise overview of exactly what patent protection entails; and (3) highlight important implications of IP for research and the researcher.

## 2 Benefits of IP protection for scientific research(ers)

The prime reason that should motivate researchers to be engaged in the development of IPR is that it is very rewarding to see a technology, originating from their own lab, developed into a final product that ultimately benefits patients.

Secondary to this, there is the potential for financial income, both for their research group and for them personally, even though the chance of substantial revenues accruing from any given patent application is typically low. Generally speaking, only a small minority of patented inventions will generate a significant return, and even then the amounts concerned are small in comparison to the institute’s total R&D budget. There are however good opportunities for funding of invention-related research, as alluded to in section 1. With respect to personal remuneration, the majority of research institutes has IP guidelines that allow individual inventors to receive a certain share of the revenues received by the institution. This income is generally being obtained through licensing of patents to a commercial entity or through the sale of shares held by the institute and/or the inventor in the spin-off company in which the invention is further developed.

A third advantage for academics that are actively involved in developing IP, is that such activities are becoming increasingly better appreciated by research institutions and are often used as a criterion to rate academics when promotions or tenure positions are to be decided upon.

Not only translational or clinical researchers may think of getting engaged in the development of IP. The example of the invention of the polymerase chain reaction shows that also fundamental researchers should keep an open eye to what possible exploitable inventions may come forth from their projects.

### 2.1 Role of the knowledge transfer office

For researchers the KTO serves as an important intermediary for contact with industry and patent lawyers. Most researchers are not trained in IP law, business development, and other business-related aspects of science. Therefore, KTO personnel who are trained in these aspects can take over much unfamiliar work from the researcher. In addition, the KTO can guide the researcher through negotiations with industry and/or the setting-up of a spin-off company, and perform most of the paperwork involved (e.g. filing patent applications, drafting confidentiality- and license agreements). In addition, some KTOs have funds to support essential proof-of-concept research studies and can issue pre-seed grants, allowing researchers to hire outside expertise when starting a company, which includes building a business plan or perform a freedom-to-operate analysis. Such an analysis is important to identify any existing IP that covers (part of) the intended product, and that could block the commercial development if no reasonable license can be negotiated with the owner of such IP.

Involving KTO business development personnel in all negotiations concerning research collaborations with industry is highly recommended, as this often results in better financial deals, including regular overhead costs in the budget, while retaining institutional IP rights to the results of the research, two aspects that often are of little or no concern to the researcher. Legal staff members screen incoming contracts and draft their own agreements, wherein particular attention is paid to the institution’s right to publish the results of its project without requiring permission from the contract partner, limiting the institution’s potential liability, and ensuring there is no conflict with pre-existing contracts (e.g., the same IP rights being provided to more than one party). This all requires little input from the researcher during drafting and finalizing the contracts.

The researcher is often involved in the initial contacts with potential licensees, which usually occur through links that already exist between the researchers and their counterparts within industry, i.e. by previous collaborative research projects. Furthermore, scientists play a crucial role in discussions with potential partners for a collaboration or license agreement, because they know their technology best and are very often a good advocate for their own invention.

As will be addressed in more detail in section 2, the patenting process also requires input from the inventors at several points in time, as their role with respect to the scientific content is indispensable. This is especially the case during the writing and editing of the first draft of the patent application and when responding to office actions.

When an inventor chooses to be involved in spinning-off an invention into a new company, the actual process of setting up the spin-off company and the paperwork involved can be time consuming, particularly when the process involves negotiations regarding the division of the company’s shares. In this regard KTOs abide by clear institutional policies on these issues.

Furthermore, when negotiating contracts for a research collaboration (sponsored research agreements (SRAs), material transfer agreements (MTAs) and confidentiality disclosure agreements (CDAs)) or when the research project is in its early stages, researchers can stay away from IP-related details and instead focus on their research, if they so prefer.

## 3 From invention to patent

‘Intellectual property rights’ is a term used to describe a variety of legal rights for different types of creations of the mind. IP rights provide owners the right to exclude others from commercializing their creations. Depending on the type of IP, rights must be applied for or are automatically established. Patents require that the object of protection is an ‘invention’ and patent rights are established by filing a patent application. Although notoriously difficult to define in a positive way, an invention can be regarded as a solution for a technical problem. Other forms of IPR, such as copyright, trademarks etc., are less relevant for research institutions. Therefore, we focus on the patent rights in this paper.

The patent system was put in place to balance the interests of the inventor and society at large: while the inventor is granted 20-year exclusive use of the invention, the underlying information is disclosed to the public, allowing others to build on the invention and create new innovations. The exclusivity provided by a patent creates a monopoly position for patent exploitation by the patent holder, which is essential since, as mentioned in the introduction, the development of products (e.g., new drugs) requires large investments. Companies typically only make such large investments if they have a reasonable chance of recouping such investment in the long run by exclusive marketing of the drug. Drugs on which the patent protection expires are quickly copied by generic pharmaceutical companies that can repeat the most essential clinical studies required for market approval at a fraction of the costs of the original drug development trajectory or often only have to show pharmacokinetic bioequivalence and the pharmaceutical quality of their product. The availability of such ‘generics’ causes the price of the drug to drop dramatically. This mechanism does not only apply to expensive drug development, but to most other industries, especially those based on technology including but not limited to telecom, medical devices, engineering, and biotech.

Important to note is that patents allow their owners to block others from using the invention without permission, but do not automatically provide them with the right to produce, sell, etc. products that are based on their invention. For instance, exploitation of a patented invention may infringe other patent rights, or national regulations may forbid exploitation (e.g. patents on atomic weapons can be applied for, but production of such weapons is not allowed).

### 3.1 Patentable inventions

Inventions need not be very complex or even clever to be patentable. In our experience, many researchers make several inventions throughout their career. However, it requires a certain awareness from researchers to let the invention “surface” and bring the invention to the attention of the KTO. In general, as long as an invention is novel, not obvious, has an industrial application, it can be patented. An invention can be a substance (e.g., drug), composition (e.g., formulation), a device, or a method (e.g., production, purification). For pharmaceutical compounds and compositions, further categories of patentable inventions exist, classified as compounds or compositions for use in a treatment (if never used before in a treatment) or in a new therapeutic application. These categories of inventions (especially the latter) can be of interest for university hospitals, as they can enable patent protection for existing drugs, even if the particular compound is already patented itself, when it has been repurposed in new therapies. Such a “second medical use patent” can be particularly attractive to the company already selling the drug.

As an example of a simple invention: back in 1989 at the Academic Medical Center in Amsterdam, two clinical virologists thought of a way to purify DNA from tissue material, which was based on the very simple discovery that DNA binds to glass beads at a certain pH and is released again at a different pH [2]. This method was patented and developed into DNA isolation kits, which have been used in molecular biological and biomedical research all over the world for more than 20 years now.

Next, we briefly explain three patentability criteria, i.e. novelty, inventive step, and industrial applicability.

**Figure 2:**
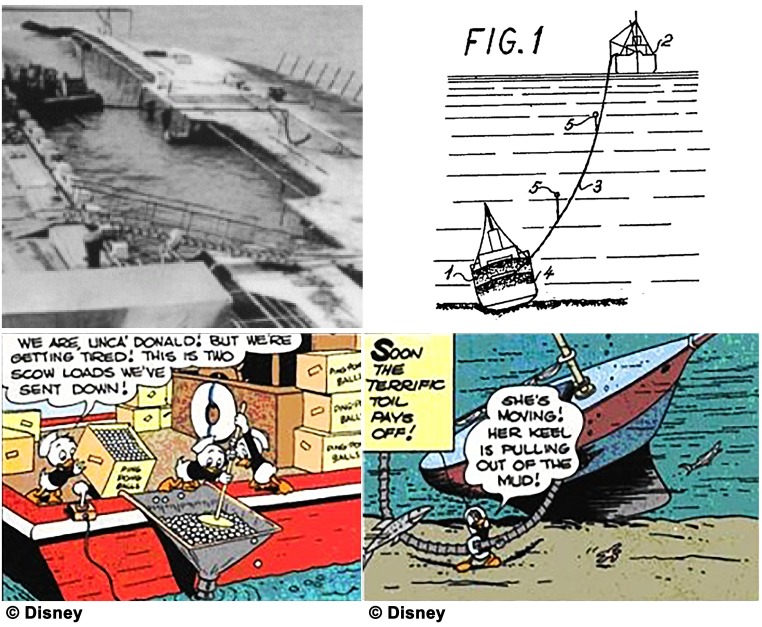
(Top panels) In 1964 a Dutch patent NL6514306A was filed based on the idea by the Danish inventor Krayer that sunken ships could more easily be salvaged by using air-filled balls to increase their buoyancy (the top right panel was taken from [3]; the top left panel can be found in [4]). (Bottom panels) The patent was not granted, as the invention had already been disclosed in a 1949 Donald Duck episode, entitled ‘The Sunken Yacht’. Interestingly, the popular TV show Mythbusters recently confirmed the feasibility of the approach taken by Donald and his nephews (https://www.youtube.com/watch?v=lKKu0DA5lvM). Permission to use the images in the bottom row were granted by Disney under a copyright license.

***Novelty:*** an invention can only be patented if it has not been described or disclosed before anywhere in the public domain. The rules regarding novelty are very strict, meaning that not only scientific articles, abstracts, and presentations at scientific conferences may be harmful, but also disclosures on personal websites, during conversations with external parties (even late at night in a bar!), or even in comic books (Figure 2).

***Inventive step:*** an invention may be novel, but not considered to contain an inventive step. An invention should not be obvious to someone ‘skilled in the art’, meaning that a person working in the same field would not come up with the same invention by using common general knowledge or by using known information particular to that field. Clearly, this requirement cannot be assessed as straightforwardly as the novelty condition. In practice, if there is no clear lack of an inventive step, then convincing the patent examiner (who eventually decides on whether a patent is granted or not) of the inventive step of that particular invention is a matter of trying to find convincing arguments. The determination whether an invention contains an inventive step requires the input of a patent attorney.

***Industrial applicability:*** This criterion is in practice of little importance. All inventions that have industrial applications, are eligible for patenting. This is the case for the vast majority of filed patent applications. In exceptional cases, for instance when an invention appeared contrary to the laws of physics (such as a perpetual motion machine or cold fusion), patents were refused based on the lack of industrial application.

Further to the patentability criteria mentioned above, there are several formal requirements needed for the disclosure of the invention. One of these formal requirements is the requirement of support for the invention: The invention must be supported by the disclosure in enough details to enable a person skilled in the art to reproduce the invention. This can be an obstacle, especially for pharmaceutical inventions. An extreme example of a lack of support is an invention wherein a compound is claimed for use in the treatment of a disease, wherein the compound has yet to be identified.

### 3.2 Obstacles for patent protection in academic settings

#### 3.2.1 Tension between publishing and patenting

Naturally, researchers’ highest priority is publishing and presenting research results in public. This conflicts with patent protection which requires absolute novelty of an invention.

While a scientist must take precautions in order to avoid loss of patentability, simple practices can be adopted to guarantee novelty protection while permitting proper and timely scientific exchange.

Submission of abstracts and manuscripts before patenting their content is possible, as long as filing of the patent application occurs before the publication date. In fact, abstracts for conferences and manuscripts submitted for publication are usually kept confidential during the review process and only when they are finally published is their content no longer considered novel. What is often not known, is that the writing and filing of a patent application can be done simultaneously with the process of submitting the scientific results for publication and therefore usually does not delay publication. In fact, a nearly finished manuscript provides an ideal basis for a patent application. If necessary, a patent attorney can draft and file a patent application on the basis of a finished manuscript in a matter of days. This is a much shorter time than that required for a scientific publication to be published.

Besides scientific publications, other disclosure situations which may lead to loss of novelty are student thesis discussions, oral presentations, or even simple scientific exchange with colleagues. However, absolute secrecy is not always required when disclosing results at presentations given within the same institution, i.e., only to people that have the same employer as the inventors. Such presentations are not harmful for the novelty of an invention, as long as the people in the audience are (made) aware that they should not disclose the content of the presentation elsewhere. Similarly, grant proposals are not considered to be a disclosure, however one should be cautious, since when the grant is awarded, some grant providers publish (part of) the application, e.g. on their websites.

Sometimes, it may be necessary to discuss the invention with others (for example with researchers from other universities or companies). In order to prevent that such discussion may be regarded as public, and become prior art for the invention, confidentiality can be arranged by means of an agreement (CDA or non-disclosure agreement (NDA)). Having a CDA in place allows a researcher to freely discuss the invention, as long as it can be traced back which party contributed which information. However it should be noted that it can be quite difficult having to prove in court that the party to which the confidential information was disclosed, is in breach of the CDA, once a dispute arises. The general advice therefore is not to disclose the invention in full detail, if it is only covered by a CDA. CDAs can be executed within a week if necessary, and are generally handled by the KTO. A good example of initially friendly scientific discussions that culminated in very expensive and complex legal battles on the IP was the case of CRISPR/cas9 [5].

#### 3.2.2 Inventorship

Scientists are not always aware of rights of the inventor and the applicant. It is important to underscore that the definition of an “inventor” may be different from the definition of e.g. principal investigator in the conventional academic hierarchy. In patent law, an inventor is a person or a group of individuals who have conceptually contributed to the claims described in the invention disclosure. Facilitating the reduction of the invention to practice is, in most cases, not sufficient to qualify as an inventor. This means that a semi-involved department head, who has furnished the funding, lab infrastructure, and technicians and who has provided the (potential) inventor with a job and salary, does not qualify for inventorship if he has not contributed intellectually to the specific claims of the patent. This is different from co-authorship of such a professional of a scientific publication. For political and judicial reasons, it is of paramount importance that (potential) inventors properly document all IP-related proceedings to abrogate false claims being made by those not entitled.

Readers should note that in almost all countries the research institution that employs the inventor is the applicant of the patent application by law and/or institute job regulations and therefore retains ownership of the patent, Exceptions include Sweden and Italy, where the inventors own their inventions under the “professor’s privilege” [6]. In 1980 the United States passed what is widely considered landmark legislation, the Bayh-Dole Act, which granted recipients of federal R&D funds the right to patent their inventions. Inventors have the right to be mentioned on the patent application as inventors.

### 3.3 Phases of a patent

**Figure 3:**
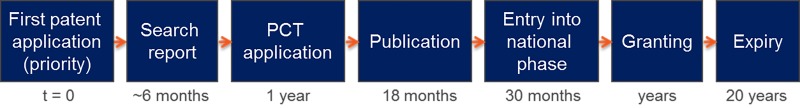
Typical patent timeline from application until expiry (20 years from the filing date of the PCT application).

The different steps and timelines of a typical patent application and granting procedure are illustrated in Figure 3 and addressed in sections 3.3.1 through 3.3.4.

#### 3.3.1 Patent filing

Especially within universities, the budget for filing and maintaining patents is usually limited (see below for a rough overview of the costs). Therefore, the decision to patent a certain invention is not taken lightly and usually involves assessment of the patentability criteria by a qualified patent attorney as well as critical commercial assessment of the business case by the KTO. Critical questions that should be raised include: is the invention really providing a solution for a relevant problem? Is the market for the invention sufficiently large to attract the attention of a company? Is it possible to convince that company to license the technology ahead of time and to develop the product or, alternatively, consider setting up a spin-off company for its further development? Who are the competitors in the potential market?

Once the decision to file a patent application has been made, the next step is choosing the best moment for filing. Filing as soon as possible minimizes the risk that someone else will come up with the same invention and file a patent earlier. The patent system is based on the first to file principle, meaning that even though a person may have invented something earlier, the first person filing a patent on the invention is entitled to the patent. Another reason to file quickly may be that the inventor has already submitted a journal article, which may be accepted for publication any time soon or will be presenting the invention at a conference at short notice.

**Figure 4:**
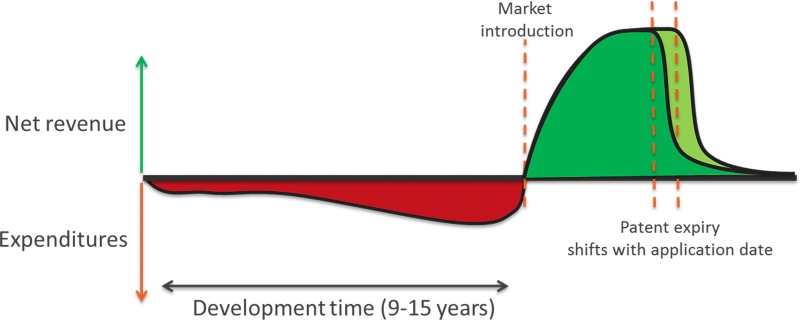
(Too) early filing of a patent application can be costly as most revenues are made at the end of the life time of the patent. Revenues collapse after patent expiry due to the introduction of generic alternatives by competitors.

If there is no pressing need to file, there are good reasons for postponing the filing of the application. Firstly, as soon as a patent has been filed, all time limits which are dependent on the filing date, i.e. the expiry date of the patent, are fixed. Usually, most revenues are generated at the end of the patent lifetime, whereas most patent and development costs are spent in the early phases (Figure 4). This is a compelling argument for filing the patent as late as possible. Secondly, the scope of patent protection of many biomedical patent applications is limited by the amount of technical evidence presented. For instance, if a certain compound is claimed for use in the treatment of a novel disease, there may be similar compounds which have the same effect. However, if supportive data are missing, it may be more difficult to obtain a broad protection for a whole class of compounds, especially in countries where post-filing evidence is not accepted. Furthermore, the inclusion of in vivo data proves the efficacy of a compound in a treatment, which is a requirement for the granting of a medical use claim. Postponing the first filing date may thus be necessary to allow the gathering of such data, providing the institute with a better chance its investment in the patent will lead to a return.

The only way to add new data to an existing invention is by filing a new application, exploiting the so called “patent priority right” (see also 3.3.2). Therefore, usually a new application is filed one year after the first (priority) application which contains the additional data. After this subsequent patent application, no new data maybe added. Therefore, if it is uncertain whether one year will be sufficient time to generate the necessary data (e.g., in vivo proof of efficacy of a new drug), it is better to postpone the filing. The filing of a patent application can be done in virtually all countries of the world, although some restrictions may apply due to the nationality of the inventor(s). European entities usually opt for filing a European application at the European Patent Office (EPO), which provides an examination report to the applicant regarding the novelty, inventive step, and industrial applicability, generally within six months of filing. US applicants usually file a patent at the US Patent & Trade Office (USPTO). The filing date of the first application (the priority date) of a European or US application may be claimed within one year, for instance by filing a subsequent PCT application (see section 3.3.2).

The process of evaluating inventions typically involves the following steps:

- The inventor reports the invention to the KTO staff. In discussions with the KTO, the technical and commercial perspectives of the invention and its potential patentability are addressed.

- If after this discussion the KTO concludes that the invention may be worth patenting, the researcher is asked to fill out a so-called invention disclosure form (IDF), which is used by most institutions. In this document the researcher describes the invention in detail, what problem it is designed to solve, and lists all the documents that resemble the invention the most, and/or the articles that inspired the inventor(s) to make the invention. Furthermore, scientists explain the invention and, when needed, which additional experiments need to be conducted to prove that the invention is working as proposed. As input for the commercial assessment by the KTO, the inventors provide their own insight into the market potential of the invention. Usually, information is provided on how many patients suffer from this particular disease, what the current solution(s) for the problem cost, and which companies might be interested in marketing the eventual new invention. Importantly, a list of (anticipated) scientific disclosures and of potentially conflicting agreements is provided. At this stage, it may turn out that an invention has already been disclosed by the inventors in full before the KTO was contacted, or given away under a contract such as an MTA. This is an indication that raising IP awareness among academics remains a continuing task of the KTO.

- If the information provided in the IDF does not contain any “deal breakers”, staff members of the KTO assess the potential value of the invention and may instruct a patent attorney to get an opinion on the patentability of the invention.

- If following these assessments no obstacles have been identified, the KTO will decide to file a patent application, and initiate the discussion between the researcher, the KTO, and a patent attorney who will draft the patent application.

During the writing of the patent application, the patent attorney typically takes the lead. Preferably, the writing of the patent application starts with formulating the claims associated with the invention, usually based on a nearly final manuscript on the invention that is about to be submitted to a scientific journal. The inventor(s) will be asked to review the application 1-2 times to answer questions by the patent attorney and to provide further input and corrections, after which the attorney files the patent application. The entire procedure can be done in a few weeks, and therefore will usually not interfere with the usual course of research in most cases or with publication as alluded to in section 1.

In case the KTO decides against filing a patent application, some institutions allow inventors to file an application at their own cost and risk.

#### 3.3.2 PCTphase

After the initial provisional patent application, there is a right (called a “priority right”) to file within one year a subsequent patent application for the same invention. For determining novelty and inventive step, the first filing date will be used. In most cases, this right is used to file an international patent application. An international application is a patent application filed under the Patent Cooperation Treaty (PCT), a treaty between more than 160 countries of the world to acknowledge the priority of each other’s first patent application. A PCT application offers the applicant a way to postpone any decision about filing separate patent applications in individual countries until 30 months from the first filing.

As the costs of a PCT application are considerable, priority applications that receive a negative search report and/or those that after the first year still have insufficient experimental data are often discontinued at this point. Most applications, however, do enter the PCT phase. If relevant, additional results that provide further support for the claims can be added. When filing a PCT application, the KTO will discuss with the inventors 1-3 months in advance whether any results obtained since the first filing could be used to strengthen the original application, and should therefore be added by the patent attorney. Inventors are asked to provide a detailed description of the results and the materials and methods used to obtain these results, and to carefully read through and provide comments on the (drafts of the) PCT application written by the patent attorney.

#### 3.3.3 National phase

At 30 or 31 months (depending on the territory) after the first filing of the patent application or 18 months after the ensuing PCT filing, the applicant needs to choose in which countries/regions the application should be granted. The aim is to get the application granted in primarily those jurisdictions that are considered to represent a major market for the product(s) derived from the invention, or where the chief competitors are located. Depending on the number ofjurisdictions elected this can be very expensive. The costs consist of filing fees, patent attorney fees (for each foreign jurisdiction a separate intermediary agent is required), and translation costs, applicable to e.g. Chinese and Japanese applications.

After entering the national phase, the patent application is further examined in each jurisdiction by an examiner of the national patent office. It is very unusual for a patent to be granted without any comments by a local examiner. Often examiners disagree with the scope of the claims, requiring the applicant to either come up with counter-arguments or reduce the breadth of the claims. These discussions are commonly done in writing, in so-called ‘office actions’. The desired outcome of these office actions is the granting of the patent. However, it is possible that none of the claims are accepted and the granting of the patent is denied. Another outcome may be that the claims that are finally granted are worthless from a commercial point of view and the decision has to be made by the applicant to drop the application. It may also happen that a competitor or potential infringer of the patent opposes the patent in an attempt to have it revoked, e.g., by arguing that the invention should not have been granted for lack of novelty, inventive step, etc.

The role of the researchers during this phase is limited to assisting the patent attorney in the defense of the claims by responding to any arguments the opponents may have.

#### 3.3.4 Costs of filing a patent

Costs of the initial and PCT filings consist mainly of official filing fees and attorney fees. Depending on the size of the application (number of pages and drawings), country of filing, and the attorney used, the costs can vary. In the national phase (section 3.3.3) translation costs are to be considered. The estimations below (in Euros) are based on typical commercial rates. Costs can be substantially lower when using an in-house patent attorney.

**Table:** 

First (priority) filing:	~7-15K
Second (PCT) filing:	~7-10K
National filings:	~3-7K/jurisdiction
Office actions:	~1-3K/office action

Other costs for granting/annual fees: up to 100-150K, for a patent filed in ~5-10 jurisdictions until expiry of the patent; if the patent is challenged in court, litigation costs can be substantial (millions).

## 4 The role of the knowledge transfer office

University KTOs started to blossom in the US after the US government approved the Bayh-Dole act in 1980, which provided academic institutions with the ownership of the inventions of their employees. In Europe, with few exceptions such as at the Catholic University Leuven and several UK universities, KTOs were established much later, starting around the year 2000.

KTOs are primarily service organizations and should strive to minimize the efforts of researchers in line with Oxford University KTO’s motto that “the key to success is to help researchers who want help commercializing the results of their research” [7]. Their main task is to enable and promote the transfer of technologies developed at universities (new medicines, medical devices, diagnostics, and outside the medical field, more durable energy solutions, better communication technology, and software, to name a few), to parties that can create impact and value, i.e., parties that can develop and bring products to the market that will affect people’s daily lives. Good examples of the impact IP protection has made can be found in the Impact Report 2015 published by ASTP-Proton, the EU association of technology transfer managers [8]. KTOs function by providing solid expertise regarding the identification, protection, and commercialization of IP, negotiating licensing and industrial collaboration contracts, and assisting with all related matters such as confidential disclosures and material transfer agreements (CDA and MTA) as well as spin-off company formation and acquisition of funding for IP-related projects.

Over the past few years many grant-providing organizations, including the EU and several charitable funds, have been putting more and more emphasis on the (societal) impact of the requested project in the overall grant approval process. So, in addition to their activities enabling impact of university technologies, KTOs are becoming increasingly involved in providing applicants with input for the impact paragraph needed in such grants, serving as an extension of the institutional grant office. Furthermore, the shrinking budgets of governmental and charity research funding are leading to many researchers considering the option of participating in public-private collaborations, often stimulated by governmental policy. Such collaborations require contract negotiations, for which the KTO is the most suitable body within the university. The KTO can also help in creating partnerships between industry and individual researchers, departments, and dedicated organizations that can provide expertise that is relevant to industry-academic collaborations (e.g., well-documented biobanks, preclinical contract research organizations, and clinical test sites).

The KTO staff generally consists of licensing officers (also referred to as business developers or technology transfer managers) and contract lawyers. A licensing officer typically has a background with scientific and industrial experience, has access to relevant industrial networks, and understands the niceties of patents and agreements. Together with contract lawyers, who are experts on the legal issues found within IP-related agreements, they negotiate with their counterparts at the industrial entity, the terms of the licensing deals and collaborative projects. Licensing officers also are the main point of contact within a KTO for personnel involved in the IP process, such as patent attorneys.

Some KTOs employ an in-house patent attorney(s). Clearly, there needs to be sufficient cases to handle and thus a certain size of the institution and KTO is required, in order to justify the hiring of an patent attorney. The main advantage of an in-house patent attorney is that fewer patent applications tend to be filed at a too-premature stage due to the involvement of the patent attorney early in the process. Consequently, the total costs of the patent portfolio can be reduced when the attorney writes and files most applications and handles the office actions him/herself.

Ideally, the KTO is well-known throughout the whole institution. As new people enter the institution every year, it is a continuous task of the KTO to advertise itself within the institution and to create awareness and educate scientists in the field of patenting and technology transfer. Through regular meetings with principal investigators, presentations during departmental meetings and specific workshops, as well as through an attractive website (http://www.ixa.nl/en/home.html), KTOs can display their expertise and work on increasing their visibility while raising awareness about the utility and necessity of IP in general.

Finally, managing expectations is also an important task of the KTO. Researchers should realize that the main reason for securing IP rights is to increase the chance that their invention is developed into a commercial product. The chance of obtaining significant revenues (for their department or in private) is very small, as only few patents lead to blockbuster products.

## 5 Intellectual property and spin-off companies: implications for the researcher

In most cases academic inventions are at a very early stage and still require further research and development before an existing company would show interest in licensing the technology. Setting up a spin-off company may then be a promising alternative to direct licensing to an external company, to move the technology outside the academic environment, which commonly hampers the further development of the invention due to limited amounts of time and focus. If a particular technology requires a more industrial approach for its further development (e.g., validation steps, pre-clinical testing, CE marking), funding options may also be better for the spin-off company than for a department at the academic institution. The KTO may then opt for incorporating a spin-off company. As the inventor’s contributions are crucial, especially in the early, formative phase of the spin-off, such a decision is always taken together with the inventor.

The role of the researcher in the spin-off company may vary. Often the researcher will be responsible for all science-related affairs within the company, and will not serve as a general manager or chief executive officer (CEO). This particularly applies to the initial stages of a start-up, as most researchers lack the required entrepreneurial skills to lead a company and attract larger investments needed for e.g., clinical trials. Only few individual researchers remain successful when it comes to managing their start-up company in the long run. Consequently, the ideal role assumed by most involved researchers is that of a chief scientific officer (CSO). Researchers may also choose to entirely refrain from an active management role in the company and only become a member of the scientific advisory board.

There are no generally accepted remuneration schemes for researchers who participate in a spin-off company. There are differences between countries and within countries, as well as between different institutions. In The Netherlands for instance, each university has its own guidelines/rules for allowing inventors to accept shares in the spin-off.

The IP, which generally is owned by the institution is licensed to the spin-off, either as part of the total package in exchange for which the institution receives shares in the spin-off company, or money set against future royalties in the form of upfront fees, milestone payments, and a royalty percentage on the revenues the spin-off generates. Combinations of shares and royalties are also possible.

Any income the institution generates from IP licensing is generally shared between the institution, the inventor’s department, and the inventor. Depending on the institution’s policy, the inventor may not be entitled to a share of the institution’s income when he/she is a shareholder in the spin-off.

## 6 Concluding remarks

In this paper we have tried to inform scientists at academic institutions about the most important aspects of IP. In our experience, many scientists do not place much emphasis on securing the IP rights on an invention, or are unfamiliar with the process, which prompted us to write this paper. The underlying thought process, however, is essentially simple and applies to most situations in translational and clinical research. The chances that an invention will ultimately reach the target user are strikingly slim without valid IP protection on the invention. The low probability of a non-patented product reaching the market is due to the magnitude of the costs associated with the development and implementation trajectory, which is basically unavoidable for most inventions. Importantly, these costs are typically too high to be fully covered by the majority of public research funding agencies. We have had a patent agency perform a cost estimate for the technology referenced in [9], which yielded an estimate of total costs of approximately 15 million Euros. Even the largest consortium grants from Brussels could not entirely cover these costs.

Securing an IP position on an invention opens numerous avenues that could financially facilitate the development and launch of a new product or service. The exclusivity and safeguards of a strong IP position automatically create a win-win situation around a viable and sustainable business model for all parties involved. For example, angel investors and venture capitalists demand return on investment (ROI) for their infusion of high-risk capital backing, which scientists need to develop their invention. IP largely secures ROI because it allows companies to block direct competitors and thus obtain and retain an exclusive market share for the lifetime of the patent, once the product has passed the preclinical and clinical trial phases and has obtained marketing approval from the regulatory agencies (e.g. FDA/EMA). The same need for a solid IP position applies to companies interested in licensing the invention. Companies depend on significant revenue streams over longer periods to cover the costs of product development and to make a profit, and hence generally do not invest in products not protected by IP.

Researchers should realize that the main reason for securing IP rights is to increase the chance that their invention is developed into a product, and also that only very few patents lead to significant revenues.

In the final analysis, if clinical and translational researchers want their invention to help patients, they should opt for securing IP rights on their invention. In our opinion and experience, the (potential) benefits, as summarized in this paper, clearly outweigh the (potential) cons. Moreover, the path to eventual clinical application is not a solo adventure. Luckily, ample infrastructure and support in the form of KTOs is present in most academic institutions to help researchers with matters for which they have no expertise.
